# Urinary Levels of Sirtuin-1, π-Glutathione S-Transferase, and Mitochondrial DNA in Maize Farmer Occupationally Exposed to Herbicide

**DOI:** 10.3390/toxics10050252

**Published:** 2022-05-17

**Authors:** Supakit Khacha-ananda, Unchisa Intayoung, Klintean Wunnapuk, Kanyapak Kohsuwan, Pitchayuth Srisai, Ratana Sapbamrer

**Affiliations:** 1 Department of Forensic Medicine, Faculty of Medicine, Chiang Mai University, 110 Inthawarorot Road, Sri Phum, Muang, Chiang Mai 50200, Thailand; ieynoghk@gmail.com (U.I.); klintean.w@cmu.ac.th (K.W.); kanyapak_koh@cmu.ac.th (K.K.); 2 Research Center in Bioresources for Agriculture, Industry and Medicine, Chiang Mai University, 239, Huay Kaew Road, Muang, Chiang Mai 50200, Thailand; 3 Graduate School, Chiang Mai University, Chiang Mai 50200, Thailand; pitchayuth.sr@gmail.com; 4 Department of Community Medicine, Faculty of Medicine, Chiang Mai University, Chiang Mai 50200, Thailand; ratana.sapbamrer@cmu.ac.th

**Keywords:** herbicide, farmer, mitochondrial DNA

## Abstract

Epidemiologic studies have suggested an association between agrochemical exposure and risk of renal injury. Farmers face great risks to developing adverse effects. The most appropriate biomarker related to renal injury needs to be developed to encounter earlier detection. We aim to study the association between early renal biomarker and occupational herbicide exposure in maize farmers, Thailand. Sixty-four farmers were recruited and interviewed concerning demographic data, herbicide usage, and protective behavior. Two spot urines before (pre-work task) and after (post-work task) herbicide spraying were collected. To estimate the intensity of exposure, the cumulative herbicide exposure intensity index (cumulative EII) was also calculated from activities on the farm, type of personal protective equipment (PPE) use, as well as duration and frequency of exposure. Four candidate renal biomarkers including π-GST, sirtuin-1, mitochondrial DNA (mtDNA) were measured. Most subjects were male and mostly sprayed three herbicides including glyphosate-based herbicides (GBH), paraquat, and 2,4-dichlorophenoxyacetic acid (2,4-D). A type of activity in farm was mixing and spraying herbicide. Our finding demonstrated no statistical significance of all biomarker levels between pre- and post-work task urine. To compare between single and cocktail use of herbicide, there was no statistical difference in all biomarker levels between pre- and post-work task urine. However, the urinary mtDNA seems to be increased in post-work task urine. Moreover, the cumulative EII was strongly associated with change in mtDNA content in both *ND-1* and *COX-3* gene. The possibility of urinary mtDNA as a valuable biomarker was promising as a noninvasive benchmark for early detection of the risk of developing renal injury from herbicide exposure.

## 1. Introduction

Chronic kidney disease of unknown etiology (CKDu) has been globally reported to be associated with agricultural workers without traditional causes such as diabetes, hypertension, primary glomerular disease, or obstructive nephropathy [1]. The exposure of agrochemical substances especially herbicide is one of the possible causes to initiate a kidney injury [2]. Previous research showed that the highest prevalence of CKDu was found in Sri Lankan farmers who were occupationally exposed to glyphosate. In addition, the topsoil and lakes in this area were contaminated with glyphosate as well [3,4]. The possible mechanism of renal injury-induced glyphosate was based on an induction of oxidative stress resulting in renal cell damage. The abnormality of renal function in animal studies was found after exposure with glyphosate-based commercial formulation of Roundup herbicide, whereas glyphosate alone had no effect on exposed animals [5]. Moreover, the findings of proximal tubular epithelial vacuolar degeneration, and abnormal serum creatinine were observed in patients who suicidally ingested glyphosate-based herbicide (GBH) [6]. Evidence indicates that acute kidney injury (AKI) survivors have a risk factor to develop the progressive CKD and even end-stage renal disease (ESRD). Moreover, AKI is associated with significant morbidity and subsequent CKD development [7]. The injured proximal tubules of kidney resulting from inflammation and fibrosis contributed to the underlying of AKI to CKD progression. The mitochondrial dysfunction in tubular epithelial cells was also classified as a crucial contributor of AKI to CKD progression [8]. Therefore, the identification of early AKI is very important.

Thailand is one of southeast Asian countries exporting high-value agricultural products such as rice, sugarcane, cassava, corn, and tobacco, as well as rubbers. The agricultural area covers approximately 47% of the land and agricultural workers is about 38% of Thai population [9]. A previous study reported that Thailand had a high prevalence of kidney disease and the number of kidney failures increased 18-fold from 10 years ago [10]. Aekplakorn et al., 2021 found that farmers who lived in a rural area were one independent risk factor for renal injury. Two reasons to support this finding were (a) dehydrate condition during working and (b) pesticides and heavy metals usage [11]. In the case of pesticide exposure, six pesticides including 2,4-dichlorophenoxyacetic acid (2,4-D), paraquat dichloride, captan, cypermethrin, glyphosate, and 1,2-Dibromo-3-chloropropane (DBCP) have been reported to be strongly associated with AKI [12]. The pesticides and their metabolites directly affected the renal cells by oxidative stress-mediated tissue injury. Moreover, the upregulation of oxidative stress and proinflammatory signaling linked with a hallmark of kidney disease were observed in mice exposed to glyphosate [13]. Although the conventional markers such as serum creatinine (SCr), cystatin C, blood urea nitrogen (BUN), estimated GFR (eGFR), and albumin to creatinine ratio (ACR) could be used for diagnosis, the markers significantly elevated in late period of disease. Therefore, the surrogate biomarker with high degree of sensitivity and specificity are being explored as favorable tools for early diagnosis of the disease [14].

Based on mechanism of herbicide-induced renal injury, a kidney injury was caused from free radical generated from herbicide. It is believed that renal mitochondrial injury is probably caused after exposure with glyphosate. To support this hypothesis, several researchers discovered that the decrease of mitochondrial potential resulting from oxidative stress was caused from the exposure with glyphosate-based commercial formulation of Roundup [15]. Moreover, the epithelial injury in proximal tubules and mitochondrial toxicity were developed after ingestion of glyphosate-based herbicide (GBH). The renal cytosolic and mitochondrial substances might be released into urine after tubular injury [6]. These substances could be considered as surrogate damage biomarker indicating structural damage from glyphosate and responsible for the progression of renal injury. Several damage biomarkers such as neutrophil gelatinase-associated lipocalin (NGAL), kidney injury molecule-1 (KIM-1), and interleukin-18 (IL-18) have been studied in AKI, however these biomarkers tended to be specific with some clinical setting and gave some limitations. For instance, NGAL was specific with sepsis, chronic kidney disease, and urinary tract infection. The elevation of KIM-1 was observed in the setting of chronic proteinuria and inflammatory diseases. Interleukin-18 could not be predicted AKI in adults. In addition, some biomarker levels remain elevated for a period of time [16]. Hence, the aim of this study is to discover the candidate renal biomarkers for prediction of acute kidney injury among farmers who are occupationally exposed to herbicide.

## 2. Materials and Methods

### 2.1. Ethical Approval

This study was carried out in accordance with the Ethics Committee of the Faculty of Medicine, Chiang Mai University, Thailand (No. 105/2021). All subjects provided informed written consent and interviewed about demographic data including herbicide usage in farm and behavior to wear personal protective equipment (PPE).

### 2.2. Location and Population

**Figure 1:**
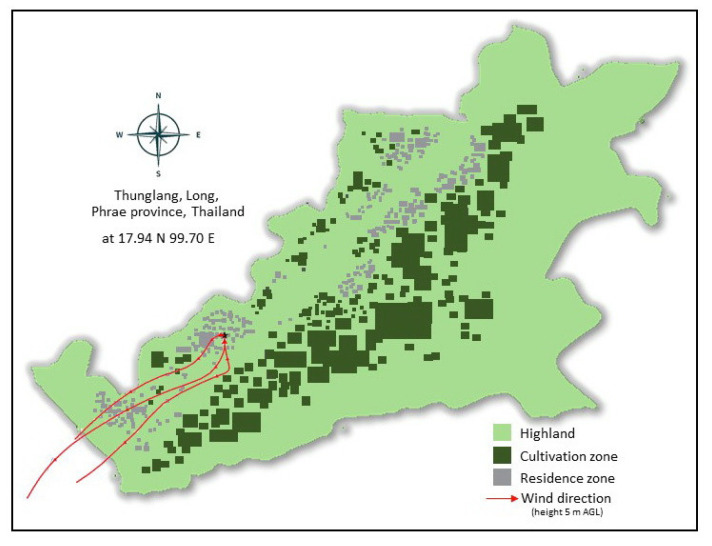
The geography of Thung Lang subdistrict, Long district, Phrae province, Thailand. This area is mainly mountainous and plain where residential and agricultural zone.

The study area was conducted in an agricultural community in Thung Lang subdistrict, Long district, Phrae province, Thailand where is situated at a latitude of 17°57′30″ N and a longitude of 99°46′21″ E in the northern part of Thailand. The topography of this area is mainly mountainous (80%) and only 20% is plain where residential and agricultural zone is about 11% and 9%, respectively. The agricultural zone covers the northern and southern parts of residential zone. As a result, the agricultural zone is typically located near or in the same area as residential zone (Figure 1). Approximately 80 percent of the population in this study area work on agricultural activities. Majority of the workforce is engaged in agricultural task and animal husbandry. The farmers grow rice, maize, and orange throughout the year. The agricultural season for maize production cover two periods (November–February and May–August). Many activities of farmer in the maize production are to landfill preparation with herbicide spraying, seed sowing, fertilization, and harvesting. The frequently herbicide use is GBH, 2,4-D, paraquat, and atrazine [17]. Therefore, herbicides have been sprayed heavily to eliminate weed to be cheaper and more effective than hand weeding and cultivation.

The experiment was designed in longitudinal follow-up study using pre-exposure of participants as a control where the same participants are followed over a period of time. Briefly, we recruited 64 individuals, 25–80 years of age, using herbicide in farms during the study period. They have never been diagnosed with kidney disease, diabetes, and gout. The questionnaires were administered by interviewed face-to-face to sex, age, smoking, alcohol related habits, characterize their work, working hours on the farm, year of work, type of herbicide usage, and use of PPE.

### 2.3. Urine Sample Collections

A morning spot urine sample before herbicide spraying (pre-work task) and after herbicide spraying (post-work task) were collected. The urine samples in the pre-work task were taken at the beginning of the period of herbicide application (approximately 08.00 a.m. in the morning of Day 1). The post-work task samples were taken at the morning of next two days (48 h after beginning of pre-work collection). All samples were stored at −20 °C until analysis.

### 2.4. Cumulative Herbicide Exposure Index Intensity (Cumulative EII)

To estimate the herbicide exposure intensity during working on farm, herbicide exposure index intensity (EII) was calculated according to Dosemeci et al. (2002) [18]. The data from questionnaire such as mixing status, applicator repairing status, application method, PPE usage during spraying were used for calculation as follows:

EII = (Mixing status + Application method + Repair status) × Personal protective equipment

where:

Mixing status: never mixing (score = 0), and mixed (score = 9)
Application method: does not apply (score = 0), aerial aircraft (score = 1), distribute tablets (score = 1), application in furrow (score = 2), boom tractor (score = 3), backpack (score = 8), and hand spray (score = 9)
Repair status: dose not repair (score = 0), and repair (score = 2)

Personal protective equipment (PPE) is classified according to types of PPE usage as shown in Table 1:

Then, a cumulative herbicide exposure intensity index was subsequently calculated as follows:

Cumulative herbicide exposure intensity index (Cumulative EII) = EII × Duration × Frequency

where:

EII = the exposure intensity index
Duration = the duration of exposure for the number of days applied
Frequency = the frequency of exposure in the number of hours of applications per day

### 2.5. Quantification of Urinary Creatinine

The quantification of urinary creatinine level in pre- and post-work task urine was determined by automated chemistry analyzer. The urine samples were sent to the Associated Medical Sciences Clinical Center, Chiang Mai University to analyte creatinine level using automatic clinical chemistry analyzer (ARCHITECTTM ci8200, Abbott, IL, USA). The urinary creatinine level was expressed as mg/dL and used for urinary biomarker normalization.

### 2.6. Quantification of Urinary Microalbumin

The determination of urinary microalbumin in pre- and post-work task urine was quantified by automated chemistry analyzer. The urine samples were sent to the Associated Medical Sciences Clinical Center, Chiang Mai University to analyte microalbumin level using automatic clinical chemistry analyzer (Mindray BS-380, Mindray, China). The detection range was between 4–300 mg/L. The urinary microalbumin level was expressed as mg/L. In addition, the microalbumin-creatinine ratio (ACR) was calculated and expressed as mg/g Cr.

### 2.7. Quantification of Urinary π-GST

The determination of urinary π-GST in pre- and post-work task urine was quantified by human glutathione S transferases-pi (GST-Pi) ELISA Kit according to the manufacturer’s instructions (MyBioSource, San Diego, CA, USA). One-hundred microliters of urine sample was added into wells which were pre-coated with monoclonal antibody to π-GST. After incubation for 90 min, the substrate was added into the well and the plate was incubated at 37 °C for 45 min. The stop reagent was then added into the wells. The color reaction was measured at a wavelength of 450 nm by microplate reader (Synergy H4; BioTek Instruments, Inc., Winooski, VT, USA). The concentration of π-GST in the samples was calculated to compare with the standard curve. The amount of π-GST was expressed as nanogram per milligram of creatinine (ng/mg Cr).

### 2.8. Quantification of Urinary Sirtuin-1

The urinary sirtuin-1 protein in pre- and post-work task urine was detected by human sirtuin 1 (SIRT1) ELISA Kit according to the manufacturer’s instructions (MyBioSource, San Diego, CA, USA). One-hundred microliters of urine sample was added into wells which were pre-coated with monoclonal antibody to sirtuin-1. Then, the plate was washed, and the biotinylated antibodies were added into the well. The avidin-peroxidase conjugates were also added. The color reaction was developed by the reaction between TMB substrates and peroxidase enzyme. Finally, the reaction was stopped by stop solution. The color intensity was measured at a wavelength of 450 nm using a microplate reader (Synergy H4; BioTek Instruments, Inc., Winooski, VT, USA). The concentration of urinary sirtuin-1 was calculated to compare with the standard curve and expressed as nanogram per milligram of creatinine (ng/mg Cr).

### 2.9. Quantification of Mitochondrial DNA (mtDNA)

The proportion of mtDNA copy was determined by quantitative real-time polymerase chain reaction (qRT-PCR). Two specific regions on mtDNA were selected including *NADH-ubiquinone oxidoreductase chain 1* (*ND-1*) and *cytochrome c oxidase subunit III* (*COX-3*). One milliliter of urine was mixed with absolute ethanol. After centrifugation, the mtDNA was extracted from urine by lysis buffer containing of 100 mM NaCl, 10 mM Tris-HCl, 0.5% SDS pH8, and 20 µL of proteinase K (20 mg/mL). After incubation, the mixture was centrifuged at 10,000 rpm for 3 min. The aqueous layer was transferred to a new tube and phenol was added (Merck, Darmstadt, Germany). After centrifugation, the aqueous layer was transferred to a new tube and the mixture between phenol and chloroform was added. After collection aqueous layer by centrifugation, the chloroform and isopropanol were subsequently added into a tube. The pellet was collected by centrifugation and washed by 70% ethanol. Finally, the pellet was resuspended with nuclease-free water [19]. Total DNA concentration was measured by NanoDrop Spectrophotometer (NanoDrop™ 8000 Spectrophotometer, Thermo Scientific, Waltham, MA, USA). The target regions on mtDNA were amplified by Applied Biosystems 7500 FAST Real-Time PCR System (Thermo Scientific, Waltham, MA, USA). The amplification condition was as follows: 95 °C for 10 min, 95 °C for 15 s, 40 cycles of 60 °C for 1 min and finally 72 °C for 30 s. The number of PCR cycle or threshold cycle (Ct) was recorded [20]. The Ct is inversely proportional to the amount of mtDNA in urine sample. Two sequences of *ND-1* (forward 5′-TCATCTGTAGGCTCATTC-3′ and reverse 5′-GCGATCCATATAGTCACT-3′) and *COX-3* (forward 5′-AGTCACCCTAGCCATCATTC-TACT-3′ and reverse 5′-GGAGTAATCAGAGGTGTTCTTGTGT-3′) primers were used [21].

### 2.10. Statistical Analysis

Descriptive statistic was used to describe the demographic data of the research population. The Kolmogorov-Smirnov test was used to test normal distribution of data. The level of biomarkers which was non-normally distributed data was represented as median and 95% confidence interval (95% CI). Wilcoxon matched pairs signed rank test was performed to compare the biomarker level between pre- and post-work task urine. Mann Whitney test was used to compare the urinary biomarker level between single and cocktail use of herbicide. Spearman correlation was analyzed to show a correlation among all biomarkers. Finally, the association between independent variable and change in urinary biomarkers was determined by a linear regression model. A *p*-value less than 0.05 is statistically significant.

## 3. Results

The demographic characteristics are shown in Table 2. Most subjects were male (62.50%) with age between 25–76 years. The farmer had approximately 12–39 years of farming experience. Most subject was non-drinker (56.25%) and non-smoker (79.69%). The subjects frequently wore PPE such as glove, boots, and facial mask during their working. A type of activity in farm was mixing and spraying herbicide. None of the subject worked in repair of herbicide applicator. Approximately 97% of subjects used a high-pressure lance sprayer and 2 of 64 also used herbicide backpack sprayer. Moreover, three of herbicides including GBH, paraquat, and 2,4-D were widely used in study area. The herbicide was practically diluted in water (1 part of herbicide to 200 parts of water) and poured into 200-litre spray tanks. Approximately 20% of farmer sprayed herbicide at 6–15 tanks per day. During this study period, all farmers sprayed herbicide for two consecutive days. Average time spraying on Day 1 and 2 was 6.31 and 5.84 h, respectively. The cumulative EII which was calculated from activities in farm, type of PPE use, as well as duration and frequency of exposure of all subjects was ranged from 10.80–316.80. To represent physiology of renal status in our subjected, the average urinary microalbumin in pre- and post-work task sample was 8.18 mg/L (min–max: 0–95.1 mg/L) and 10.27 mg/L (min–max: 0–106.6 mg/L). We found that a significant increase in microalbumin in post-work task urine, compared with that in pre-work task urine. In addition, the microalbumin-creatinine ratio (ACR) was calculated. The result showed that average ACR in pre- and post-work task sample was 20.94 mg/g Cr (min–max: 0–341.19 mg/g Cr) and 26.12 mg/g Cr (min–max: 0–431.96 mg/g Cr), respectively.

**Figure 2:**
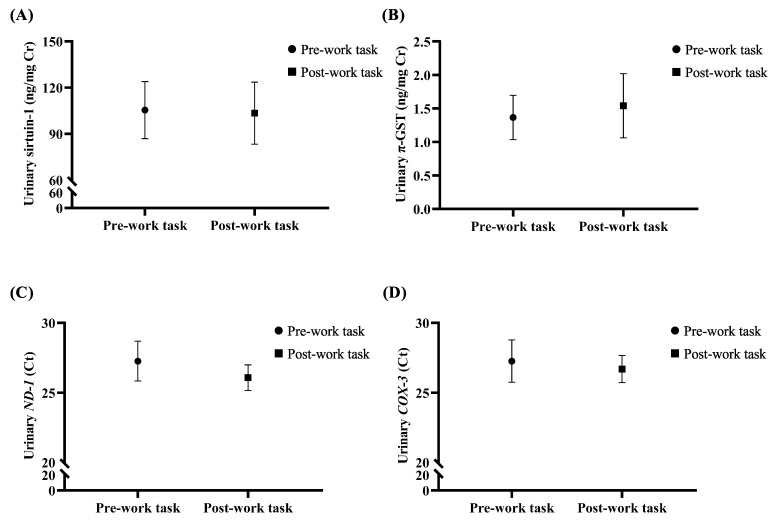
Comparison of urinary biomarkers (**A**) sirtuin-1, (**B**) π-glutathione S-transferase (π-GST), (**C**) *NADH-ubiquinone oxidoreductase chain 1* (*ND-1*), and (**D**) *cytochrome c oxidase subunit III* (*COX-3*) between pre- and post-work task urine sample. The data represented as median and 95% confidence interval. ng: nanogram; mg: milligram; Cr: creatinine; Ct: threshold cycle.

The level of urinary biomarkers including sirtuin-1, π-GST, *ND-1* and *COX-3* was compared between pre- and post-work task urine. The result demonstrated that the Ct of urinary *ND-1* (post-task urine: median = 25.64 and 95% CI = 23.67–28.34 vs. pre-task urine: median = 27.75 and 95% CI = 23.40–30.03) and *COX-3* (post-task urine: median = 26.67 and 95% CI = 23.85–29.08 vs. pre-task urine: median = 27.56 and 95%CI = 22.92–30.32) in post-work task urine tended to decrease compared to pre-work task urine. However, no statistical significance of all biomarker level between pre- and post-work task urine was observed (Figure 2).

**Figure 3:**
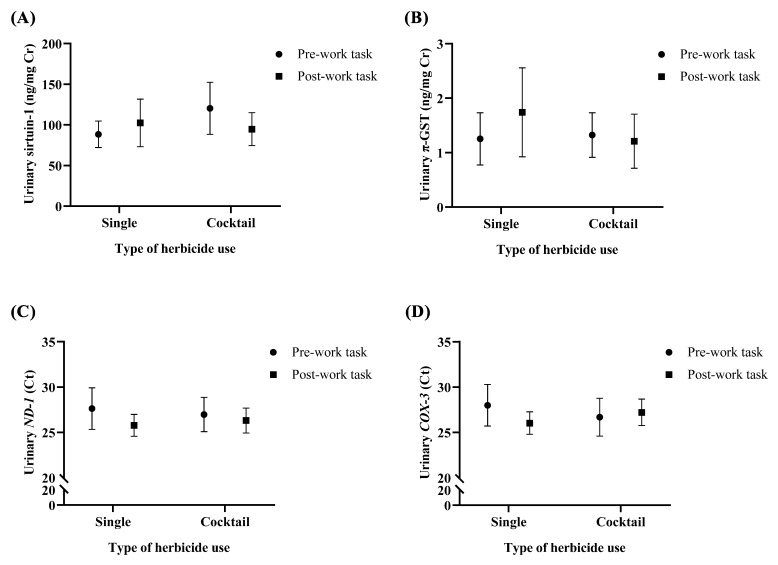
Comparison of urinary biomarkers (**A**) sirtuin-1, (**B**) π-glutathione S-transferase (π-GST), (**C**) *NADH-ubiquinone oxidoreductase chain 1* (*ND-1*), and (**D**) *cytochrome c oxidase subunit III* (*COX-3*) between pre- and post-work task sample of farmers who sprayed single or cocktail use of herbicide. The data represented as median and 95% confidence interval. ng: nanogram; mg: milligram; Cr: creatinine; Ct: threshold cycle.

To compare a biomarker level between type of herbicide usage, the subjects were divided into 2 groups: single (*n* = 28) and cocktail (*n* = 36) use of herbicide. The single of herbicide usage was farmers who only sprayed GBH in farm. The cocktail use of herbicide was farmers who sprayed mixed herbicides between GBH and paraquat and 2,4-D. The Ct of urinary *ND-1* (single use of herbicide in post-task urine: median = 25.90 and 95% CI = 23.72–27.56 vs. pre-task urine: median = 27.93 and 95% CI = 22.57–30.20 and cocktail use of herbicide in post-task urine: median = 25.61 and 95% CI = 23.38–28.91 vs. pre-task urine: median = 27.27 and 95% CI = 23.68–29.95) and *COX-3* (single use of herbicide in post-task urine: median = 25.74 and 95% CI = 23.80–28.04 vs. pre-task urine: median = 29.00 and 95% CI = 22.79–31.13 and cocktail use of herbicide in post-task urine: median = 27.06 and 95% CI = 23.85–30.32 vs. pre-task urine: median = 27.18 and 95% CI = 22.92–30.23) in post-work task urine tended to decrease compared to pre-work task urine in farmers who sprayed both single and cocktail use of herbicide. Moreover, the level of sirtuin-1 and π-GST slightly increased in post-work task urine among farmers who sprayed single type of herbicide. However, no statistical significance of all biomarker level between pre- and post-work task urine was observed in farmers who sprayed both single and cocktail use of herbicide (Figure 3).

To study a correlation between urinary renal injury biomarker, Spearman correlation analysis was analyzed. First, the C_t_ of mtDNA was calculated into urinary DNA concentration (pg/ng creatinine) by using DNA standard curve. A standard curve was generated by serial dilution of control DNA 9948 (Qiagen, Hilden, Germany). Then, the biomarker level of both urine samples (pre- and post-work task) was transformed into change in biomarker level. The change in biomarker level derived from delta (delta = level in post-work task urine–level in pre-work task urine) was calculated in all urine subjects. The result showed that the delta sirtuin-1 level increased correspondingly with *COX-3* (r = 0.308, *p* = 0.015). Moreover, we found a high degree of correlation between the delta *ND-1* and *COX-3* (r = 0.604, *p* < 0.001). In addition, Spearman correlation between urinary microalbumin and *ND-1* was 0.453 (*p* < 0.001). In addition, Spearman correlation between urinary microalbumin and *COX-3* was 0.257 (*p* = 0.044). Spearman correlation coefficients are presented in Table 3.

To study influencing factor on the level of renal injury biomarker, the association between independent variable and change in biomarker level was analyzed. The result is presented in Table 4. We found that the cumulative EII was positively associated with change in *ND-1* and *COX-3*. It could be described that an increase in one unit in cumulative EII showed an association with an increase in change of *ND-1* and *COX-3* by approximately 0.619 and 0.287 units, respectively. In addition, the increase in one unit in year of farming experience significantly associated with an increase in change of *COX-3* by 1.177 unit. Furthermore, a high positive association between the cumulative EII and change in *ND-1* (B = 1.183) as well as *COX-3* (B = 0.524) was presented in the urine of farmers who sprayed cocktail use of herbicides.

## 4. Discussion

It is known that an occupational exposure of herbicide exerts negative effects on humans. Nephrotoxic acute kidney injury is one of the adverse effects caused from an exposure of herbicides in Asia and the Pacific region. Mohamed et al., 2015 summarized that paraquat and glyphosate had common causes of AKI with the incidence more than 50% [22]. The oxidative stress and uncoupling of oxidative phosphorylation have been proposed as a primary mechanism of these two herbicides to induce kidney injury [23,24]. The uncoupling of oxidative stress in mitochondria affects mitochondrial function and integrity. The damaged mitochondria and mitochondrial dysfunction contributed to the progression of kidney dysfunction [25]. According to mechanism of toxicity, farmers face a high risk to develop acute or chronic renal injury after spraying a mixture of herbicides on the farm. Hence, the discovery of early renal injury biomarkers that rely on the mechanism of toxicity of herbicide was potentially useful for health surveillance and protection of farmers. We aimed to discover the renal biomarker related to renal injury via mitochondrial toxicity from occupational exposure of herbicide.

This study investigated the change in renal injury biomarkers in maize farmers over 48 h of the herbicide spraying with different type of herbicides. Two spot urines were collected before herbicide spraying (pre-work task urine sample) and 48 h after herbicide spraying (post-work task urine sample). During farm working, they generally used three types of herbicides such as GBH, paraquat, and 2,4-D. Most farmers worked in preparation and spraying of herbicide in maize farm. Damalas and Koutroubas, 2016 noted that mixing and loading task are the section which farmers have a high risk to be directly exposed to herbicide due to spill and splash without safety training and the use of PPE [26]. We found that the farmers wore PPE such as gloves, boots, and facial mask. To estimate herbicide exposure, the cumulative EII was calculated. Although the direct measurement of herbicide concentration in biological specimens is critical for the exposure assessment, there is a challenge due to limited availability of biomarkers of exposure, the practical difficulties and costs in large populations, as well as multiple routes of exposure. In addition, it is often not feasible for short biological half-lives where the timing of measurements around periods of pesticide use is critical [27,28]. Several factors affect the exposure level such as duration of work in farm, mixing of pesticide, habits of spraying, equipment of protection, and habits of hygiene in personal work [29]. As a result, indirect methods of estimation are more frequently used. A few studies have discovered the algorithm for estimation of exposure intensity which was based on questionnaires collecting job titles, occupational history, personal protective equipment worn, type of activity on the farm, method of application, as well as duration and frequency of working time [18]. Several studies showed strongly significant correlations between exposure intensity index and the concentration of urinary pesticide metabolite in the applicators [30,31,32]. Importantly, the finding from all studies obviously indicated that algorithm exposure intensity scores based on self-reported data are significantly related to measured levels. In addition, this algorithm has been used to estimate exposure intensity in several publications [29,33,34,35].

Due to the lack of non-exposure group, the urinary microalbumin and ACR in urine sample were determined. These biomarkers have been used for detection of kidney damage and end stage renal disease (ESRD). In addition, they were proposed to show a strong candidate for the prediction of renal risk from many diseases which presented of functional and/or structural renal abnormalities [36]. Moreover, urinary microalbumin was found to be considered as markers for early detection of nephropathy with significant correlation with serum cystatin C [37]. El-Ashmawy et al., 2014 demonstrated the significant correlation between microalbumin and KIM-1 in type II diabetic patients [38]. As our finding, the average of urinary microalbumin in post-task urine was significantly increased. The report of Ji et al., 2020 demonstrated that the average of urinary microalbumin level in normal and early renal injury patients was 4.0 mg/L (min–max: 2–12.8 mg/L) in normal and 11.2 mg/L (min–max: 2–123 mg/L) [39]. Our result showed that ACR level in our subjects were classified as normal to moderately increased of kidney disease according to National Kidney foundation. Hence, it could be assumed that the slight increase of urinary microalbumin was correlated with the mild renal injury. Subsequently, four renal biomarkers were selected to determine early kidney injury. Two biomarkers (sirtuin-1 and π-GST) represented the renal injury biomarker. The others (*ND-1* and *COX-3*) also represented as a specific biomarker indicating renal mitochondrial toxicity.

Although the level of studied biomarkers was not significantly different between pre- and post-work task urine, the C_t_ of two mitochondrial DNA region (*ND-1* and *COX-3*) seems to decrease after herbicide spraying. In the case of sirtuin-1, it is a protein in the class of histone deacetylases. The function of this protein is involved in cell proliferation, DNA repair, and mitochondrial energy homeostasis [40]. Two of seven sirtuin proteins (sirtuin-1 and sirtuin-3) have been widely studied about renal injury. Sirtuin-1 related with homeostasis of renal cells, whereas sirtuin-3 is also related with the regulation of ATP synthesis [41]. The high expression of sirtuin-1 was found in the proximal tubule which acts to preserve mitochondrial functional integrity [42]. A previous study reported that *sirtuin-1* expression was associated with the oxidative stress response. The overexpression of *sirtuin-1* promoted the expression of *Nrf2* which is regulatory genes of superoxide dismutase, glutathione, catalase and heme oxygenase-1 in paraquat-induced injury in mouse model [43]. Although we found no significant difference in sirtuin-1 level, this detected level in our study could not be classified as an abnormal level due to no prior study about reference range of urinary sirtuin-1 level in healthy subjects.

The glutathione S-transferase (GST) in the group of α-GST and π-GST is a renal-specific protein. It can be found in epithelial cells of proximal and distal tubule [44]. This protein could be used as a biomarker for tubular damage since it was released into urine after renal injury [45]. Many publications showed the usefulness of GST in a variety of clinical manifestations for example toxic substance-induced nephrotoxicity, diabetic patients with varying degrees of albuminuria, and proteinuria with normal glomerular filtration rate (GFR) [46,47,48]. The clinical study in glomerular diseases and proteinuria found that increased urinary excretion of π-GST were observed in patients with renal failure, whereas α-GST were found in patients with a well-preserved renal function [49]. The biomarker level in non-exposure subjects could not be performed in our study, however the biomarker level of our population was compared with the reference level in healthy subjects and renal disease cases from other publications. Only one report demonstrated the average of urinary sirtuin-1 level in Turkish people which was 3.29 (min–max 1.42–50) ng/mL [50]. In addition, our study found the average urinary sirtuin-1 level in pre- and post-work task sample was 52.53 ± 31.42 and 48.84 ± 39.72 ng/mL. In addition, an average of urinary π-GST in our farmers was 1.42 and 1.55 ng/mg creatinine of pre- and post-work task urine, respectively. Minimum and maximum value of urinary π-GST in our study ranged from 0–6.27 and 0–7.58 ng/mg creatinine. According to Brüning et al., 1999, they reported an average of urinary π-GST in German healthy adult being 2.3 ± 0.65 ng/mg creatinine, whereas this marker level in subjects who were exposed with substance-induced kidney disease was 6.0 ± 3.3 ng/mg creatinine [51]. In addition, the median of urinary π-GST in Polish healthy volunteer with no kidney dysfunction was 3.24 (interquartile range = 2.18–4.12) ng/mg creatinine [52]. The average urinary π-GST level among healthy subjects, normoalbuminuria, microalbuminuria, and macroalbuminuria was 0.63 (range 0–2.7), 1.35 (range 0–30.6), 1.8 (range 0–33.3), 1.17 (range 0–44.1) ng/mg creatinine. This finding reported that the level of π-GST increased across the normo-, micro- and macroalbuminuria groups [53]. Although we found no statistical significance in π-GST level, it could be assumed that some farmers in our study had the exceeded level of urinary π-GST indicating an earlier phase of kidney injury.

Mitochondrial DNA (mtDNA), the Ct of *ND-1* and *COX-3* was compared. Due to an inverse proportion between Ct and mtDNA content, the decrease of Ct in both genes of post-work task urine could be described that the mtDNA content possibly increased. Basically, the difference in 1 cycle of qRT-PCR means a 2-fold difference in DNA copies. To further ascertain the fold-change in mtDNA, we found that the different median of Ct between post- and pre-task urine was about 2 and 1 cycle for *ND-1* and *COX-3*, respectively. Therefore, it represented a median fold-change of 4- and 2-fold at *ND-1* and *COX-3* copies in post-work task urine, respectively. *ND-1* and *COX-3* encodes the protein for mitochondrial respiratory chain and subunits of complex-I, respectively [54]. The renal cell enriched with mitochondria since the renal tubular cells especially proximal tubule, distal convoluted tubule, and connecting segments have the highest oxygen consumption for reabsorption and excretion processes [55]. The depletion of energy in mitochondria and increase of reactive oxygen species promoted the mitochondrial swelling and fragmentation. The disruption of mitochondrial structure triggered the release of many substances such as cytochrome c and mtDNA to activate cell death and proinflammatory danger signal, respectively [56]. In addition, the mtDNA was released from damaged mitochondrial in renal tubular cell and then activated toll-like receptor-9 to further propagate renal injury [20]. Abassi et al., 2013 reported that the urinary mtDNA was significantly correlated with urinary levels of tubular injury markers [57]. The study of urinary mtDNA level after herbicide exposure has not been previously reported. However, change in mtDNA level was found to be significant in inflammatory responses to injury in a sepsis patient. The mtDNA can be detected in urine within 24 h after renal injury [56]. Moreover, the mitochondrial injury and the level of urinary mtDNA were found to be significant with acute kidney injury in surgical critical illness patients [21].

Comparable measurements of the renal injury between farmers who used single and those using cocktail use of herbicide, showed no significance between the two groups. However, the mtDNA level was likely to be elevated in post-work task urine of both single and cocktail use of herbicides. One hypothesis explaining this finding is that the use of PPE and working time on the farm probably reduced the exposure of herbicide into the body since all participants in our study wore PPE. Moreover, an average of working time on the farm for herbicide spraying of our subjects was approximately 5–6 h per day. Consistent with Wongwichi et al., 2021, they stated that the risk of herbicide exposure in maize farmers in Thailand decreased when farmers wore PPE and sprayed herbicide on the farm for less than 5 h per day [58]. The Korean farmers wearing great number of PPE and doing more protective behaviors such as showering and changing clothes after contact with herbicide had remarkably reduced levels of oxidative biomarker resulted from herbicide toxicity [59]. The observation of Konthonbut et al., 2020 found that the decrease of dermal exposure to paraquat during working depended on type of herbicide applicator including wearing a long sleeve shirt, long pants, boots, latex gloves, and balaclava [60]. With regard to the type of herbicide application, our subjects mostly use high-pressure lance sprayer and 2 of 65 subjects also use backpack sprayer. The farmers who used backpack sprayer have been reported to have a high risk for alachlor herbicide exposure. Since this applicator generated a cloud of sprayed droplets when spraying, moreover the spillage of herbicide onto the back of farmers was found in older age of many backpack sprayers [61]. Knudsen et al., 2017 reported that the elimination half-life of glyphosate was very rapid within 3.1 h after exposure [62]. In addition, the wearing of gloves while mixing and loading herbicide greatly reduced the glyphosate concentration in urine [63]. Interestingly, participants in our study were also more likely to have detectable urinary glyphosate concentration which was lower than occupational exposure limit. It is plausible that our subjects directly exposed the low dose of herbicide while working on the farm resulting in insignificantly induced toxicity in kidney.

To demonstrate the influencing factors on renal injury biomarkers, a linear regression analysis was performed. The result demonstrated that cumulative EII was significantly associated with change in mtDNA level both *ND-1* and *COX-3* regions in all participants. The increase of cumulative EII in 1 unit is directly associated with the elevation of mtDNA both *ND-1* and *COX-3* approximately 0.619 and 0.287 unit, respectively. Hence, it was assumed that all influencing factors in cumulative EII including work task on the farm, application method, repair status, PPE use, as well as duration and frequency of spraying on the farm may have a major impact on mtDNA. Furthermore, the cumulative EII had a great association with change in mtDNA levels in urine samples from farmers who sprayed a cocktail use of herbicides. The cocktail use of herbicide in the class of glyphosate, paraquat, and 2,4-D might be synergistically exerted the effect on renal mitochondrial damage. The synergistic mechanism of two herbicides was based on the abnormal protein synthesis. The glyphosate affected the translation process of Multidrug and Toxin Extrusion 1 (MATE1) protein. So, paraquat highly accumulated in renal cells since the export of paraquat from the apical membrane of tubular cells into the tubule lumina for excretion via MATE1 was decreased [64]. Likewise, the combination of glyphosate plus 2,4-D enhanced the genetic damage in *Cnesterodon decemmaculatus* model [65]. Our research also has limitations that the lack of non-exposure group as a reference level of biomarker in healthy subjects since non-exposure group in the same anthropometric characteristics for this study area would be difficult to recruit. Most people in this area work in agricultural activities, so each family has household family members who are agriculturists. Although the main exposure of herbicide was from working, a take-home and environmental exposure was a probable route for exposure. A take-home occurs when farmers or workers fortuitously carry home herbicide residue or agrochemical substances on his or her clothing or shoes, thereby potentially exposing his or her family. Moreover, the location of a farm can be located next to a residential zone. So, the non-exposure subjects faced a risk to being exposed to pesticide from a dispersion in the environment caused by spray drift and volatilization of pesticides at the time of application or soon after. However, a further study to determine biomarker level on a control group is necessary to evaluate the impact of herbicide exposure on biomarkers. In addition, the measurement of biomarker outcomes after exposure to pesticides in only 48 h might not be enough to detect the renal injury caused from herbicide exposure. Therefore, a prolonged follow-up study is further needed to be studied.

## 5. Conclusions

The present study was designed to investigate the urinary renal injury biomarkers and the influencing factors on these biomarker levels. Our study population practically sprayed a cocktail of herbicides between GBH, paraquat, and 2,4-D with personal protective equipment. Regarding biomarker analysis, no significant change in the biomarker level in pre- and post-work task samples. However, the level of urinary mtDNA slightly increased after herbicide exposure. We found that the strong positive correlation between two of mtDNA marker (*ND-1* and *COX-3*) was significantly observed. Importantly, our finding demonstrated that there is a significant association between herbicide exposure and urinary mtDNA level among farmers exposed to herbicide especially GBH, paraquat, and 2,4-D. Overall, the level of mtDNA could be suggested to be a biomarker for adverse effect surveillance and identification of the occurrence of herbicide-associated renal injury.

## Figures and Tables

**Table 1:** Scoring for personal protective equipment usage.

Parameters	Score
Never used PPE (PPE-0)	1.0
Face shields or goggles, fabric/leather gloves, other protective clothing (PPE-1)	0.8
Cartridge respirator or gas mask, Disposable outer clothing (PPE-2)	0.7
Chemically resistant rubber gloves (PPE-3)	0.6
PPE-1 & PPE-2	0.5
PPE-1 & PPE-3	0.4
PPE-2 & PPE-3	0.3
PPE-1 & PPE-2 & PPE-3	0.1

**Table 2:** The demographic characteristics of studied population (*n* = 64).

Variable	Characteristics	Frequencies (*n*)	Percentage
Gender:	Male	40	62.5
	Female	24	37.5
Age:	≤45	15	23.44
	46–55	28	43.75
	56–65	16	25
	≥66	5	7.81
Year of farming experience	<30	23	35.94
	≥30	41	64.06
Alcohol use	Yes	28	43.75
	No	36	56.25
Tobacco use	Yes	13	20.31
	No	51	79.69
Personal protective equipment (PPE) use	Yes	63	98.44
	No	1	1.56
Type of PPE (multiple responses)	Glove	54	84.37
	Boots	63	98.46
	Facial mask	58	90.62
Activity in farm (multiple responses)	Mixing herbicide	47	73.44
	Spraying herbicide	64	100
	Repair herbicide applicator	0	0
Type of herbicide equipment	High-pressure lance sprayer	62	96.87
	Backpack sprayer	2	3.13
Volume of herbicide (tank/day)	0–5	8	12.5
	6–10	22	34.38
	11–15	21	32.81
	16–20	10	15.63
	21–25	3	4.68
Type of herbicide use	GBH	28	43.75
	GBH + paraquat + 2,4-D	36	56.25
Average time spraying (hour/day)	0–5	26	40.63
	6–10	36	56.25
	11–15	2	3.12
Day 1 time spraying (hour)	0–5	25	39.06
	6–10	37	57.81
	11–15	2	3.13
Day 2 time spraying (hour)	0–5	30	46.88
	6–10	31	48.44
	11–15	3	4.68

**Table 3:** Spearman correlation of all urinary biomarkers.

Spearman Correlation	Sirtuin-1	π-GST	*ND-1*	*COX-3*	Microalbumin
Sirtuin-1	1.000				
π-GST	0.017	1.000			
*ND-1*	0.212	0.102	1.000		
*COX-3*	0.308 *	0.056	0.604 ***	1.000	
microalbumin	0.137	−0.007	0.453 ***	0.257 *	1.000

Changes in urinary biomarker level were derived and a correlation among all biomarkers were analyzed by Spearman rank correlation analysis after outlier removal (Grubbs (Alpha = 0.05)). Statistically significant comparisons are indicated (* *p* < 0.05 and *** *p* < 0.001). π-GST: π-glutathione S-transferase; *ND-1: NADH-ubiquinone oxidoreductase chain 1*; *COX-3: cytochrome c oxidase subunit III.*

**Table 4:** The linear regression analysis in the association between influencing factors on change in urinary biomarker level.

(A) Urine sample from all subjects (*n* = 64)								
Independent variable	Sirtuin-1		π-GST		*ND-1*		*COX-3*	
	B	SE	B	SE	B	SE	B	SE
Single or cocktail use of herbicide	−41.817	24.028	−0.383	0.597	29.157	33.570	−4.841	15.019
Year of farming experience (year)	−0.031	0.946	0.013	0.023	0.787	1.321	1.177 *	0.591
Cumulative EII	−0.488	0.205	0.006	0.005	0.619 *	0.286	0.287 *	0.128
Volume of herbicide (tank)	7.336	5.305	−0.058	0.132	−7.114	7.412	−0.418	3.316
(B) Urine sample from farmers who sprayed single use of herbicide (*n* = 28)								
Independent variable	Sirtuin-1		π-GST		*ND-1*		*COX-3*	
	B	SE	B	SE	B	SE	B	SE
Year of farming experience (year)	0.036	1.348	0.031	0.040	−0.014	0.008	−0.011	0.007
Cumulative EII	−0.385	0.305	0.017	0.009	0.003	0.002	0.001	0.002
Volume of herbicide (tank)	−0.944	11.286	−0.090	0.332	−0.153	0.069	−0.082	0.061
(C) Urine sample from farmers who sprayed cocktail use of herbicide (*n* = 36)								
Independent variable	Sirtuin-1		π-GST		*ND-1*		*COX-3*	
	B	SE	B	SE	B	SE	B	SE
Year of farming experience	−0.323	1.431	0.011	0.028	0.89	2.528	2.078	1.106
Cumulative EII	−0.524	0.295	−0.004	0.006	1.183 *	0.521	0.524 *	0.228
Volume of herbicide (tank)	9.609	6.356	0.02	0.123	−12.637	11.229	−2.129	4.913

B or unstandardized regression coefficient indicated the amount of change in a dependent variable due to a change of 1 unit of independent variables. SE also indicates the standard error of regression coefficient. Statistically significant is indicated (* *p* < 0.05). π-GST: π-glutathione S-transferase; *ND-1: NADH-ubiquinone oxidoreductase chain 1*; *COX-3: cytochrome c oxidase subunit III*.

## Data Availability

Not applicable.
